# Overexpression of a Weed (*Solanum americanum*) Proteinase Inhibitor in Transgenic Tobacco Results in Increased Glandular Trichome Density and Enhanced Resistance to *Helicoverpa armigera* and *Spodoptera litura*

**DOI:** 10.3390/ijms10041896

**Published:** 2009-04-23

**Authors:** Ming Luo, Zhaoyu Wang, Huapeng Li, Kuai-Fei Xia, Yinpeng Cai, Zeng-Fu Xu

**Affiliations:** 1 State Key Laboratory of Biocontrol and Key Laboratory of Gene Engineering of the Ministry of Education, School of Life Sciences, Sun Yat-sen University, Guangzhou 510275, Guangdong, P.R. China; 2 Laboratory of Molecular Breeding of Energy Plants, Xishuangbanna Tropical Botanical Garden, Chinese Academy of Sciences, Kunming 650223, Yunnan, P.R. China

**Keywords:** Insect resistance, lepidopteran pests, protease inhibitor, Solanum americanum, trichome

## Abstract

In this study we produced transgenic tobacco plants by overexpressing a serine proteinase inhibitor gene, SaPIN2a, from the American black nightshade *Solanum americanum* under the control of the CaMV 35S promoter using *Agrobacterium tumefaciens-*mediated transformation. SaPIN2a was properly transcribed and translated as indicated by Northern blot and Western blot analyses. Functional integrity of SaPIN2a in transgenic plants was confirmed by proteinase inhibitory activity assay. Bioassays for insect resistance showed that SaPIN2a-overexpressing transgenic tobacco plants were more resistant to cotton bollworm (*Helicoverpa armigera*) and tobacco cutworm (*Spodoptera litura*) larvae, two devastating pests of important crop plants, than the control plants. Interestingly, overexpression of SaPIN2a in transgenic tobacco plants resulted in a significant increase in glandular trichome density and a promotion of trichome branching, which could also provide an additional resistance mechanism in transgenic plants against insect pests. Therefore, SaPIN2a could be used as an alternative proteinase inhibitor for the production of insect-resistant transgenic plants.

## 1. Introduction

Proteinase inhibitors (PIs) have been established as plant defense agents against insects and other pests [1,2]. The defensive role of PIs is based on the inhibitory activities towards insects’ digestive enzymes and other pathogens’ proteases involved in vital processes [3,4], which represents an important plant defense strategy against herbivores. The effectiveness of insect resistance through the overexpression of PIs in transgenic plants has been demonstrated [5–8]. Among different types of plant PIs, serine PIs were widely employed to engineer insect resistance in transgenic plants because most lepidopteran insects such as cotton bollworm (*Helicoverpa armigera*) and tobacco cutworm (*Spodoptera litura*) use serine proteinases as major digestive enzymes [9].

So far, most PI genes used for developing insect-resistant transgenic plants were obtained from crop plants [2,10], e.g. cowpea trypsin inhibitor [5], potato and tomato proteinase inhibitor II (PIN2) [6], rice cysteine PI [11], barley trypsin inhibitor [12], soybean Kunitz trypsin inhibitor [13], and maize PI [14]. Many insect pests, however, have evolved to adapt to their host plant PIs [15,16]. The adaptation of insects to PIs has resulted in the failure of some transgenic plants overexpressing PIs to resist pests [17–20]. It has been shown, however, that the PIs from non-host plant could effectively inhibit the gut proteinases of crop pests [16]. Hence the weed plants, which have evolved separately from the insect pests of crop plants, may provide a valuable alternative source of PI genes for insect-resistant transgenic crop plants. American black nightshade (*Solanum americanum*) is a weed belonging to the Solanaceae family, and a rich source of PIs [4]. We have previously cloned a serine PI, SaPIN2a, from *S. americanum* [21]. The purified native SaPIN2a significantly inhibited serine proteinases such as trypsin, chymotrypsin, and subtilisin, and also effectively inhibited midgut trypsin-like proteinases from larvae of *S. litura* and *Trichoplusia ni* [22], suggesting a potential application of SaPIN2a in the production of insect-resistant transgenic crops.

In this study, to evaluate the prospects of SaPIN2a in conferring insect-resistance to genetically modified crops, SaPIN2a has been introduced into tobacco (*Nicotiana tabacum*) via *Agrobacterium*-mediated transformation. Larvae of the noctuids *H. armigera* and *S. litura*, which are two devastating pests causing considerable economic loss world-wide to many important crops and vegetables such as cotton, tobacco, sunflower, corn, pepper and tomato plants [23,24], were fed on leaves of the transgenic plants expressing SaPIN2a. Here, we report the effects of SaPIN2a on trichome development of transgenic plants and on insect larval growth.

## 2. Results and Discussion

### 2.1. Expression of SaPIN2a in transgenic tobacco plants

**Figure 1.**
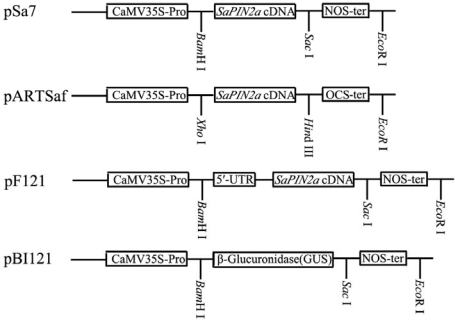
Schematic representation of the binary vectors pSa7, pARTsaf, pF121 and pBI121. The various components of the vectors are represented by boxes. CaMV35S-Pro, cauliflower mosaic virus 35S promoter; OCS-ter, octopine synthase terminator; NOS-ter, nopaline synthase terminator; 5′-UTR, SaPIN2a 5′ untranslated region.

We have previously found that SaPIN2a was not detectable in leaves and stems of transgenic lettuce plants by Western blot analysis, although a high expression of SaPIN2a mRNA was observed [25]. In transgenic nightshade, no SaPIN2a was found in leaves of the transgenic plants, whereas the amount of SaPIN2a significantly increased in the stems of transgenic nightshade plants as compared with wild-type plants [26]. Since the 5′ untranslated region (5′-UTR) of the transgenes plays an important role in determining the rate of translation in plant cells [27–29], the failure of accumulation of SaPIN2a in leaves of transgenic plants could be attributed to low translational efficiency of SaPIN2a because of the lack of the 5′-UTR of SaPIN2a in previous binary vectors pSa7 [25] and pARTSaf [26] (Figure 1). In this study, the 5′-UTR of SaPIN2a was cloned (GenBank accession No. AF174381) and used to construct a new binary vector pF121 containing the 5′-UTR and coding region of SaPIN2a cDNA (Figure 1).

**Figure 2.**
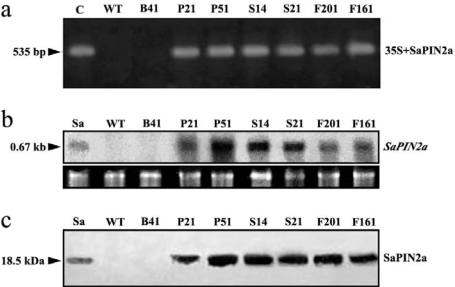
Molecular characterization of transgenic tobacco plants. (a) PCR analysis. Genomic DNA extracted from transgenic and control plants was used as template for PCR amplification with primers specific for 35S CaMV promoter and SaPIN2a cDNA. Amplified bands were indicated by an arrowhead. (b) Northern blot analysis. Total RNA (20 μg) isolated from transgenic and control plants. The blot was probed with random-primed ^32^P-labelled *SaPIN2a* cDNA. The hybridization bands corresponding to the SaPIN2a transcript (0.67 kb) are indicated by an arrowhead. Lower panel shows rRNA bands as loading controls. (c) Western blot analysis. Total proteins (50 μg) from transgenic and control plants using SaPIN2a-specific antibodies. Purified SaPIN2a (Sa, 0.4 μg) from *S. americanum* stems was used as a positive control. Cross-reacting bands (18.5 kDa) are indicated by an arrowhead. C, plasmid pARTSaf (positive control); Sa, total RNA (b) or purified SaPIN2a (c) from stems of *S. americanum* plants (positive control); WT, wild-type tobacco plants (negative control); B41, transgenic plant line transformed with pBI121 vector only (negative control); P21 and P51, transgenic plant lines transformed with pARTSaf; S14 and S21, transgenic plant lines transformed with pSa7; F201 and F161, transgenic plant lines transformed with pF121.

The pF121, together with two previously described over expression vectors pSa7 and pARTSaf containing the SaPIN2a cDNA without 5′-UTR and the control vector pBI121 (Figure 1) [25,26] were used to transform tobacco by *Agrobacterium tumefaciens*-mediated method. To determine the presence of SaPIN2a in transgenic tobacco plants, PCR analysis was carried out with primers specific for 35S CaMV promoter and SaPIN2a. The expected PCR product corresponding to the sequence of the 35S CaMV promoter and SaPIN2a was detected in transgenic plants, but not in control plants (Figure 2a). Northern blot analysis was conducted on transgenic plants to detect the presence of SaPIN2a mRNA. A SaPIN2a transcript was observed in leaves of transgenic plants (P21, P51, S14, S21, P161 and P201 in Figure 2b) and the positive control (stems of nightshade plants, Sa in Figure 2b), whereas no signal was detected in leaves of wild-type plants (WT) and the control transgenic line B41 (Figure 2b). Western blot analysis was then performed to confirm the accumulation of SaPIN2a in leaves of transgenic plants. As shown in Figure 2c, a strong SaPIN2a cross-reacting band of apparent molecular mass 18.5 kDa was detected in leaves of transgenic plant lines transformed with all three binary vectors, which is absent in wild-type plants (WT) and vector-only control transgenic line B41. Levels of SaPIN2a in transgenic plants were quantified by Western blot and estimated to be 1.2 – 1.4% of the total soluble proteins, which is in the range of previous reports [15,30]. There was no consistently significant difference observed in the expression levels of SaPIN2a in different transgenic plant lines developed with different vectors. This result suggests that the differential accumulation of transgenic SaPIN2a in different plant species including lettuce [31], nightshade [26] and tobacco (this study) may result from the specific post-translational mechanism that regulates SaPIN2a accumulation and/or traffic in different plants.

### 2.2. Proteinase inhibitory activities of transgenic SaPIN2a

**Figure 3.**
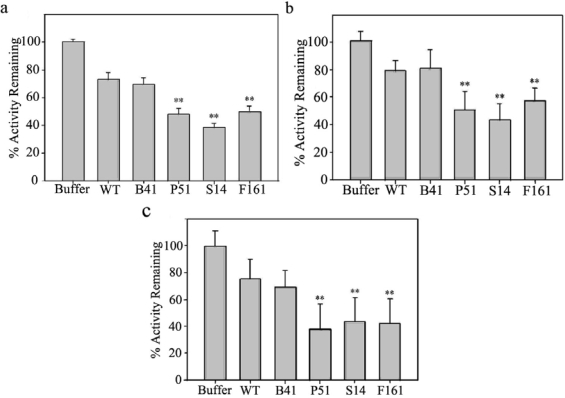
Assays of proteinase inhibitory activities of transgenic SaPIN2a. (a) Inhibition of trypsin by total soluble proteins (TSP) from leaves of tobacco plants. (b, c) Inhibition trypsin-like proteinases from *H. armigera* (b) and *S. litura* (c) midguts by TSP from leaves of tobacco plants. Buffer, extraction buffer for tobacco TSP; WT, leaves of wild-type tobacco plants (negative control); B41, transgenic plant line transformed with pBI121 vector only (negative control); P51, transgenic plant line transformed with pARTSaf; S14, transgenic plant lines transformed with pSa7; F161, transgenic plant lines transformed with pF121. Mean values plus standard errors are given. ** indicates significance at the 1% level.

We have previously shown that purified SaPIN2a from nightshade stems significantly inhibited serine proteinases such as trypsin, chymotrypsin, and subtilisin [22]. To test the functionality of SaPIN2a expressed in leaves of the transgenic tobacco plants, crude leaf extracts from transgenic and control plants were tested for inhibitory activity against bovine trypsin. As shown in Figure 3a, total protein extracts from leaves of SaPIN2a-overexpresing transgenic plants (P51, S14 and F161) showed significantly higher inhibitory activities against bovine trypsin than those from wild-type plants (WT) and vector-only control line B41.

The midgut proteinases of two devastating pests of important crop plants, *H. armigera* and *S. litura*, have been found to be primarily serine proteinases [32,33], which could be inhibited by some plant PIs *in vitro* [34,35]. We have also found purified SaPIN2a from nightshade stems effectively inhibited midgut trypsin-like proteinases from larvae of *S. litura* and *Trichoplusia ni* [22]. To further assess the inhibitory activity of transgenic SaPIN2a toward insect midgut proteinases, leaf extracts from transgenic and control plants were tested for inhibitory activity against the trypsin-like proteinases extracted from midguts of *H. armigera* and *S. litura*. Results of trypsin-like inhibitory activity assays showed leaf extracts from SaPIN2a-overexpresing transgenic plants strongly inhibited midgut trypsin-like proteinases from both *H. armigera* (Figure 3b) and *S. litura* (Figure 3c), whereas leaf extracts from control plants (WT and B41 in Figure 3b and 3c) contained little trypsin-like proteinase inhibitory activity.

### 2.3. Insect-resistant activity of transgenic SaPIN2a against H. armigera and S. litura

To determine whether SaPIN2a overexpression in transgenic tobacco plants could confer protection against insect attacks, homozygous transgenic tobacco plants were used for insect feeding trials with larvae of *H. armigera* and *S. litura*.

**Figure 4.**
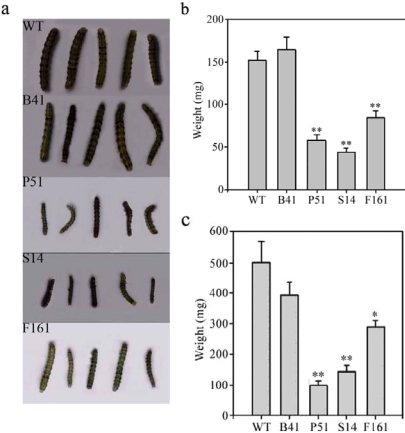
Insect bioassays of transgenic plants. Larvae were allowed to grow for 7 days on the tobacco plants. (a) *H. armigera* larvae fed on control plants (WT and B41) and SaPIN2a-overexpressing transgenic plants (P51, S14 and F161). (b) Weight of *H. armigera* inoculated on different tobacco lines. (c) Weight of *S. litura* inoculated on different tobacco lines. * indicates significance at the 5% level. ** indicates significance at the 1% level (compared with the value of WT, determined by t-test).

**Figure 5.**
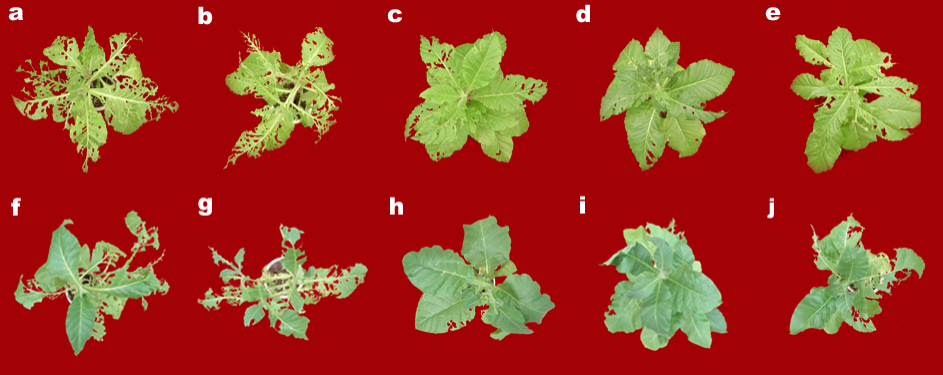
Insect resistant phenotype of SaPIN2a-overexpressing transgenic tobacco plants. Each tobacco plant was infested with 10 early second-instar larvae of *H. armigera* (a – e) or *S. litura* (f – j) for 7 days. a and f, wide-type plants; b and g, vector-only transgenic plants; c – e and h – j, SaPIN2a-overexpressing plants.

After feeding for 7 days, larvae fed on SaPIN2a-overexpresing transgenic plants (Figure 4a, P51, S14 and F161) grew significantly slower than those fed on control plants (Figure 4a, WT and B41), with a 44.3 – 71.4% and 42.3 – 80.1% reduction in the larval weight of *H. armigera* (Figure 4b) and *S. litura* (Figure 4c), respectively. More larvae died on SaPIN2a-overexpresing transgenic plants during the feeding experiment. The mortality of larvae fed on SaPIN2a-overexpresing transgenic plants was 36.7 – 50.0% for *H. armigera* and 56.7 – 86.7% for *S. litura*, whereas the mortality of larvae fed on wild-type plants was 16.7% and 43.3%, respectively (Table 1). Further feeding on transgenic leaves expressing SaPIN2a affected insect metamorphosis. The pupation rate of larvae fed on SaPIN2a-overexpresing transgenic leaves was only 26.1 – 47.6% for *H. armigera* and 50.0 – 63.9% for *S. litura*, whereas the pupation rate of larvae fed on wild-type leaves was 83.8% and 82.2%, respectively (Table 1). Figure 5 shows that wide-type plants (a, f) and vector-only transgenic plants (b, g) were severely damaged by larvae of *H. armigera* (a, b) and *S. litura* (f, g) after 7 days’ infestation, whereas transgenic plants overexpressing SaPIN2a exhibited only minor damage on the leaves (c – e, h – j). The results of these bioassays, together with the preliminary studies in transgenic lettuce [31], provide evidence that SaPIN2a, a novel PI from weed plant, can confer plants resistance to lepidopteran pests.

### 2.4. Effect of transgenic SaPIN2a on trichome development in tobacco

**Figure 6.**
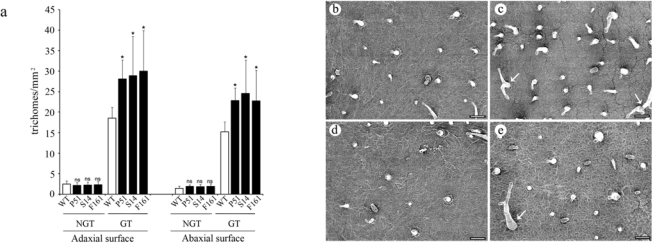
Effects of overexpression of SaPIN2a on trichome development in tobacco leaves. (a) Densities of non-glandular trichomes (NGT) and glandular trichomes (GT) on adaxial and abaxial leaf surfaces of wild-type (WT) tobacco and SaPIN2a-overexpressing transgenic lines P51, S14 and F161. Mean values plus standard errors are given. ns, not significant at the 5% level; *, significant at the 5% level. (b – e) Scanning electron micrographs of adaxial (b and c) and abaxial (d and e) leaf surfaces of wild-type tobacco (b and d) and SaPIN2a-overexpressing transgenic line P51 (c and e). Arrows in c and e indicate branched glandular trichomes. Bars, 100 μm.

We have previously shown that *S. americanum* PIN2 gene family contains two members, SaPIN2a and SaPIN2b, which are differentially expressed in plants [21,36–38]. Overexpression of SaPIN2b resulted in a significant increase in glandular trichome density and promotion of trichome branching on tobacco leaves [38]. Here, we examined the trichomes on leaves of SaPIN2a-overexpressing transgenic tobacco plants. Similar to what we found for SaPIN2b [38], more glandular trichomes, but not non-glandular trichomes, were found on both adaxial and abaxial surfaces of transgenic tobacco leaves, as compared to those on wild-type plant leaves (Figure 6). Branched glandular trichomes were also found on leaves of SaPIN2a-overexpressing transgenic plants (arrows in Figure 6c, e), which did not occur on leaves of wild-type tobacco (Figure 6b, d).

Since it has been shown that the heterogeneously expressed SaPIN2a inhibited plant endogenous serine proteases in transgenic lettuce [25], we propose that the ectopically expressed SaPIN2a in transgenic tobacco may affect trichome development through inhibiting activities of some plant proteases, like SDD1 and ALE1 [39–41], which are involved in regulation of plant epidermal differentiation and development. Glandular trichomes are specialized epidermal cells that function as a first line of defense against insect pests by various physical and chemical mechanisms [42–44] in a number of plants, including wild potato [45], potato [46], *Leonotis leonurus* [47], wild tomato [48], tomato [49–51] and pigeonpea [52]. Glandular trichomes can act as a deterrent to insect attacks, e.g. by deterring insect settling, oviposition and feeding, physical and/or chemical entrapment, repellent activities and/or toxic effects of the exudates produced by glandular trichomes [44,52–54]. We speculate, therefore, that more glandular trichomes on transgenic tobacco plants overexpressing SaPIN2a obtained in this study may contribute to the enhanced insect resistance, since some negative results were previously reported with similar PIs against *H. armigera* and *S. litura* [18,55]. Glandular trichomes could, therefore, provide an additional resistance mechanism apart from the proteinase inhibitory activity in SaPIN2a-overexpressing transgenic plants against lepidopteran pests, which is worth exploring further.

## 3. Experimental Section

### 3.1. RNA ligase-mediated rapid amplification of 5′ cDNA end (RLM-5′ RACE)

Total RNA was extracted from *S. americanum* flowers using RNeasy Plant Mini Kit (QIAGEN). The 5′ cDNA end of SaPIN2a was obtained by RLM-5′ RACE using a GeneRacer^TM^ core Kit with Superscript^TM^ II Module (Invitrogen). The SaPIN2a-gene specific primer (GSP, 5′-CTGAGCTCTTATAGCTCATCTTTGAAATAAGCAGTGGTCTTGG- 3′) was used for reverse transcription. The 5′ RACE products were cloned into pMD18-T vector (TaKaRa). The resultant plasmid p5′RACE-TV DNA was used as a template for sequencing. Sequence analysis revealed that 5′-UTR sequence of SaPIN2a was 49 nt, 5′-acccagaaaaaacaacaacaaagaaaacaaggtggagaaagcattcata-3′ (GenBank accession No. AF174381).

### 3.2. Binary vectors and plant transformation

Three binary vectors (pSa7, pARTSaf, and pF121) containing the SaPIN2a coding region (Figure 1) and the control vector pBI121 [56] were employed in this study. Binary vectors pSa7 and pARTSaf were described previously by Xu *et al.* [25] and Xie *et al.* [26], respectively. pF121 was constructed by replacing the GUS gene in pBI121 [56] with a *Bam*H I-*Sac* I fragment containing the 5′ untranslated region (5′-UTR) and coding region of SaPIN2a cDNA, which was amplified by PCR from the p5′RACE-TV described above with SaPIN2a-specific primers (forward primer 2a-full-1, 5′-GTGGATCCACCCAGAAAAAACAACAACAAAGAAGGCAA-3′ ; reverse primer 2a-full-2, 5′-ATGAGCTCTTAGAAATAAGCAGTGGTCTTGGGTTCA-3′). All binary vectors were transformed into *Agrobacterium tumefaciens* strains LBA4404 [57]. The *Agrobacterium tumefaciens*-mediated method described by Horsch *et al.* [58] was used for leaf disk transformation of tobacco (*Nicotiana tabaccum* L. cv. Xanthi). Putative transformants were selected on MS medium containing kanamycin (100 mg/l). Kanamycin-resistant plantlets were transferred to soil and grown under natural conditions (22–28 ºC, 14 h light/10 h dark) in a greenhouse.

### 3.3. PCR analysis of transgenic plants

Tobacco genomic DNA was extracted from transgenic plants and wild-type plants by the CTAB method [59]. The presence of the transgene SaPIN2a was detected by PCR amplification using the 35S CaMV promoter primer ZF56, 5′-TCCCACTATCCTTCGCAAGACCC-3′ and SaPIN2a-specific primer ZF103, 5′-GCGGATCCTTAGAAATAAGCAGTGGTCT-3′. The amplification parameters were as follows: 94 ºC for 5 min, followed by 35 cycles of 94 ºC for 1 min, 52 ºC for 1 min, 72 ºC for 1 min. Final extension time of 10 min at 72 ºC was used. PCR products were separated by a 1% agarose gel electrophoresis. Genomic DNA from wild type and vector-only control plants and plasmid pARTSaf were used as negative and positive control, respectively.

### 3.4. Northern blot and western blot analysis

Total RNA was extracted from transgenic and wild-type plants, and analyzed by Northern blots with a random-primed ^32^P-labelled *SaPIN2a* cDNA probe as previously described [21]. Total plant proteins were extracted, quantitated and analyzed by Western blot analysis according to the procedure of Xu *et al.* [25].

### 3.5. Proteinase inhibitory activity assay

Inhibition of trypsin by leaf extracts from transgenic tobacco plants (three homozygous T2 lines P51, S14, F161) was assayed following the previous procedure [25]. Fifty μg of tobacco TSP was incubated with 0.1 μg of trypsin for 3 min at 37 ºC. Wild-type and vector-only transformed plants (homozygous line B41) were used as two negative controls. Crude midgut proteins were extracted from midguts of 50 larvae of *H. armigera* and *S. litura,* respectively [22]. Inhibition of trypsin-like proteinases in the midguts of *H. armigera* and *S. litura* larvae by tobacco leaf extracts was analyzed according to the procedure of Wang *et al.* [22]. Eighty μg of tobacco TSP was incubated with 200 μg of midgut proteins from *H. armigera* or 100 μg of midgut proteins from *S. litura* for 3 min at 37 ºC.

### 3.6. Scanning electron microscopy

Trichomes on the adaxial and abaxial surface of tobacco leaves were examined with a Jeol field emission scanning electron microscope (Model JSM-6330F) as previously described [38].

### 3.7. Insect feeding trials

Three plants of each transgenic line, wild-type (WT) and vector-only control transgenic line B41 were used for insect feeding bioassays. Colonies of neonate *H. armigera* and *S. litura* larvae were supplied by Jiyuan Baiyun Industry Company Ltd. (Henan, China). Three-month old transgenic and wild-type tobacco plants (about 50 cm in height) were challenged by newly emerged larvae of *H. armigera* and *S. litura*. Ten early second-instar larvae were introduced to each tobacco plant. The larvae were allowed to feed freely on the plants that were sealed into individual insect-proof nylon net cages in a greenhouse. Seven days later, all larvae (both dead and surviving) were collected. Biomass of the surviving larvae per plant was recorded. All survival larvae collected from tobacco plants were then raised to pupation on tobacco leaves of respective transgenics and control that were replaced by fresh ones every day. *H. armigera* larvae were raised individually in Petri dishes to prevent cannibalism. The insect feeding experiment was repeated thrice and the statistical significance was calculated using Student’s *t*-test.

## 4. Conclusions

Our results demonstrate that weed plants could provide an alternative potential source of PI genes that are useful for generating insect-resistant transgenic crop plants. Overexpression of SaPIN2a, a PIN2 gene from a Solanaceae weed *S. americanum*, in transgenic tobacco plants resulted in an increase in inhibitory activities against bovine trypsin and insect midgut proteases. SaPIN2a-transgenic plants consistently showed enhanced resistance to *H. armigera* and *S. litura*. In addition, overexpression of SaPIN2a increased the density of glandular trichomes of transgenic plants, which may also contribute to the enhanced resistance to *H. armigera* and *S. litura*. These results suggested that SaPIN2a could be a promising PI for the production of insect-resistant transgenic plants.

## Figures and Tables

**Table 1.** Mortality and pupation rate of *H. armigera* and *S. litura* larvae feeding on SaPIN2a-overexpressing and control tobacco plants. Ten early second-instar larvae were introduced to each plant of transgenic lines and wild-type plants (WT). Three plants of each transgenic line and WT were used for insect feeding bioassays.

	**Mortality (%)**		**Pupation rate (%)**	
	*H. armigera*	*S. litura*	*H. armigera*	*S. litura*
WT	16.7 ± 3.3	43.3 ± 3.3	83.8 ± 4.4	82.2 ± 1.1
P51	36.7 ± 3.3 *	56.7 ± 8.8 *	47.6 ± 2.4**	63.9 ± 7.3 *
S14	50.0 ± 5.8 **	66.7 ± 6.7 *	26.1 ± 3.9**	50.0 ± 0.0 *
F161	40.0 ± 5.8 **	86.7 ± 6.7 **	32.1 ± 6.6**	33.3 ± 16.7 **

* and ** indicate significance at the 5% and 1% level, respectively.
